# Trimethoprim-Sulfamethoxazole-Induced Pancytopenia: A Case Report

**DOI:** 10.7759/cureus.75113

**Published:** 2024-12-04

**Authors:** Jaspreet Chahal, Gretchen Junko

**Affiliations:** 1 Department of Internal Medicine, Edward Via College of Osteopathic Medicine, Blacksburg, USA; 2 Department of Internal Medicine, LewisGale Medical Center, Roanoke, USA

**Keywords:** adverse effect of bactrim, bactrim, drug-induced adverse effect, drug-induced pancytopenia, myelosuppression, pancytopenia, surgical wound infection, trimethoprim-sulfamethoxazole (tmp-smx)

## Abstract

Pancytopenia is defined as a decrease in all three myeloid cell lines, usually from a precipitating factor such as an autoimmune condition, prescription drug, or several other factors. The etiology of pancytopenia can be determined through laboratory testing, a peripheral blood smear, and a thorough history and physical examination. Trimethoprim-sulfamethoxazole (TMP/SMX, also known by the brand name of Bactrim® or Septra®) is known to cause pancytopenia in rare cases. Still, it has yet to be fully explored or explained in the literature. We present a case of TMP/SMX-induced pancytopenia that swiftly resolved with cessation of the drug and repletion of platelets. This report aims to serve as an example of a severe case of TMP/SMX-induced pancytopenia, its presentation, and medical treatment in the acute care hospital setting.

## Introduction

Pancytopenia is defined as the decrease in all three myeloid cell lines: white blood cells, red blood cells, and platelets. More specifically, the definition is composed of a hemoglobin level of less than 12 g/dL in females or less than 13 g/dL in males, platelets of less than 150 x 10^3^/μL, and leukocytes of less than 4.0 x 10^3^/μL [1,2]. There are many causes of pancytopenia documented in the literature including megaloblastic anemia, aplastic anemia, acute myeloid leukemia, erythroid hyperplasia, genetic and autoimmune etiologies, pregnancy, as well as certain prescription medications [2,3]. To establish an accurate etiology of pancytopenia, a full workup must be completed, including laboratory tests and a peripheral blood smear.

Trimethoprim-sulfamethoxazole (TMP/SMX, also known by the brand name of Bactrim® or Septra®) is known to cause pancytopenia but the literature is limited, as very few cases have been reported. Those that have been reported did not reach the level of pancytopenia seen in this case report. One case report described a 62-year-old female with similar symptoms following five days of TMP/SMX usage that resulted in an ICU admission before resolution [4]. This case did cite a vitamin B12 deficiency that was not addressed at the time of the case, which could have impacted the patient’s outcome [4], as it is known that prolonged use of TMP/SMX has poorer outcomes in those with nutritional deficiencies [5]. There was also a cohort study done in Great Britain that demonstrated both hematological and dermatological adverse events with long-term TMP/SMX usage [6]. This data was collected over several years and does not demonstrate the potential acute adverse effects of the drug. In the present case report, we describe a unique case of acute severe pancytopenia induced by TMP/SMX, its presenting symptoms, treatment, and resolution.

## Case presentation

An 80-year-old male presented to the emergency department (ED) with the primary concern of a syncopal episode. He also reported nausea, vomiting, diarrhea, and lethargy for two days prior to the episode. Of note, he had undergone a partial right knee replacement two months prior, which later led to the development of a surgical site abscess. He was seen by his surgeon five days prior to his visit to the ED for this abscess, who prescribed TMP/SMX to treat the abscess. Of note, incision and drainage of the abscess were not indicated at the time due to its size.

Upon presentation to the ED, the patient’s review of systems revealed generalized weakness, decreased appetite, unintentional weight loss of 6 lbs, slight redness over his surgical site, headaches, dysphagia, nausea, non-bloody emesis, watery diarrhea, and lightheadedness over the course of five days. His initial vital signs included a blood pressure of 113/53 mmHg, heart rate of 97 bpm, temperature of 98°F, respiratory rate of 18 bpm, and an oxygen saturation of 96% on room air. Physical examination was pertinent for poor skin turgor, slow capillary refill, palatal and lower extremity petechiae, diffuse bilateral wheezes, as well as joint tenderness and erythema over the right knee surgical site.

**Figure 1:**
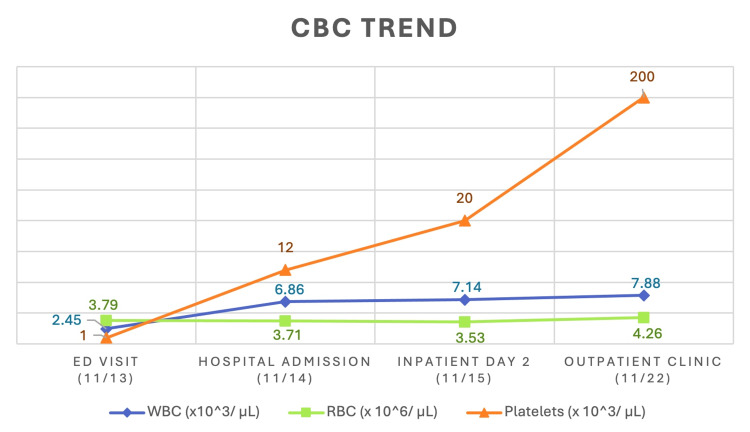
Trend of Hematologic Laboratory Values Trend of the WBC, RBC, and platelet values derived from the patient's CBC including initial values drawn in the ED, up to the one-week follow-up lab draw in the outpatient setting. WBC, white blood cells; RBC, red blood cells; CBC, complete blood count; ED, emergency department.

Initial laboratory findings in the ED showed the following: WBC of 2.45 x 10^3^/μL, RBC of 3.79 x 10^6^/μL, hemoglobin of 9 g/dL, and platelets of 1.0 x 10^3^/μL. The most clinically relevant data points are hemoglobin and platelet levels. For this patient, supportive care was provided and empiric antibiotics for possible skin infection were initiated in the ED, while the internal medicine service was consulted for admission to the hospital. The preliminary treatment plan was to discontinue TMP/SMX, administer alternative antibiotics for the surgical site infection, transfuse two units of platelets, infuse a bolus of intravenous normal saline, and administer steroids in the form of methylprednisolone (Solumedrol®, Pfizer, New York, NY) and dexamethasone (Decadron®, Bausch Health, Laval, Quebec, Canada). Although steroids are not the typical management for pancytopenia, they were initially started as they are integral in the management of immune thrombocytopenic purpura or thrombotic thrombocytopenic purpura, both of which were considered in the initial differential diagnosis. The hematology team was consulted for guidance on inpatient management as well as the criteria this patient should meet prior to discharge. The laboratory studies ordered included a coagulation panel, folate and B12 levels, bacterial and fungal cultures, peripheral blood smear, and a repeat complete blood count (CBC) with flow cytometry to assess the response to the initial treatment. The coagulation panel, B12, and folate levels resulted within normal limits, the bacterial and fungal cultures revealed no growth after 24 hours, and the peripheral blood smear did not reveal any schistocytes, platelet aggregations, or hypersegmented neutrophils. The repeat CBC 24 h later showed an improvement in the pancytopenia with a rise in WBC from 2.45 to 7.14 x 10^3^/μL, RBC level from 3.79 to 3.53 x 10^6^/μL, and platelet level from 1.0 to 20.0 x 10^3^/μL. Hemoglobin slightly increased but remained stable, showing a change from 9 g/dL to 9.3 g/dL. The trend of the patient's hematologic laboratory values, from an ED visit through an outpatient office visit, is demonstrated in Figure 1. Hemoglobin was not included in this table due to the values staying relatively stable throughout the case.

Per the hematology team’s discretion, the patient was discharged once the platelet level reached 20.0 x 10^3^/μL. On follow-up in the clinic one week later, a repeat CBC with flow cytometry was ordered as well as a serum protein electrophoresis and a hepatitis panel. The serum protein electrophoresis and hepatitis panel were within normal limits and the repeat CBC showed further improvement in the transient pancytopenia. The WBC resulted as 7.88 x 10^3^/μL, RBC resulted as 4.26 x 10^6^/μL, and platelets resulted as 200.0 x 10^3^/μL, as demonstrated in Figure 1. Hemoglobin upon outpatient visit did not show as much of a drastic change, with the third value at 9.1 g/dL. However, the patient appeared to return to baseline in terms of their appearance and review of systems. The cause of the transient pancytopenia was determined to be a reaction to the TMP/SMX, as testing did not reveal an alternate plausible etiology.

## Discussion

TMP-SMX is an antibacterial agent used to treat a variety of conditions. TMP works as an inhibitor of dihydrofolate reductase, and SMX is a structural analog of para-aminobenzoic acid. These two drugs competitively inhibit dihydropteroate synthetase in the folate synthesis pathway, which provides bactericidal effects [7]. TMP/SMX has been noted to have a large range of adverse effects, including anemia, myelosuppression, gastrointestinal symptoms, urticaria, loss of appetite, fatigue, and many more [8]. The effects of toxicity of the drug are an extension of the pharmacology, including bone marrow depression, thrombocytopenia, confusion, uremia, and more [8]. 

Few cases of TMP/SMX-induced pancytopenia have been documented in the literature. Another case was of a 62-year-old female with similar non-specific symptoms of lethargy, weakness, and fatigue after being prescribed TMP/SMX for a urinary tract infection and was hospitalized three days after finishing the five-day course. In this case, the platelet level only decreased to 50,000 k/uL and the patient decompensated and needed ICU admission [4]. Although this case was also a severe hospital course, it did not demonstrate how critical platelet levels may become due to TMP/SMX.

The mechanism by which TMP/SMX causes pancytopenia is still unknown; however, existing literature includes some explanations behind the pathophysiology. One proposed mechanism refers to drug-dependent antibodies for inducing thrombocytopenia [9]. The theory states that drug-dependent antibodies bind to epitopes on the surface of glycoproteins, but only when in the presence of the drug. However, this explanation would only be upheld if a patient is exposed to the drug for the first time and then develops the antibodies, inducing pancytopenia on the second exposure [9]. More research is needed to determine the exact mechanism by which the pancytopenia from TMP/SMX occurs. 

In the case described in this report, the patient developed symptoms that correlated with the adverse effects of TMP/SMX approximately five days after starting the antibiotic for a possible post-surgical abscess. A full workup was completed to determine other potential causes of pancytopenia, from immune thrombocytopenia to hepatitis, as liver disease is known to produce hematological manifestations [10]. Once the drug was discontinued, all three cell lines showed marked improvement, which further confirmed TMP/SMX as the likely source of the symptomology.

## Conclusions

Pancytopenia secondary to antibiotics or prescription drugs is a topic that is not well explored. This report explains a case of severe pancytopenia after utilizing an antibacterial agent, TMP/SMX. Further research is needed to develop a complete understanding of the mechanism by which TMP/SMX causes pancytopenia. However, this report highlights the unique level to which this adverse effect can occur and establishes itself as a notable reference in hospitalized cases of pancytopenia.
